# Phase II trial of neoadjuvant pemetrexed plus cisplatin followed by surgery and radiation in the treatment of pleural mesothelioma

**DOI:** 10.1186/1471-2407-13-22

**Published:** 2013-01-16

**Authors:** Rea Federico, Favaretto Adolfo, Marulli Giuseppe, Spaggiari Lorenzo, DePas Tommaso Martino, Ceribelli Anna, Paccagnella Adriano, Crivellari Gino, Russo Francesca, Ceccarelli Matteo, Kazeem Gbenga, Marchi Paolo, Facciolo Francesco

**Affiliations:** 1 Azienda Ospedaliera-Università di Padova, U.O. Chirurgia Toracica, Via Giustiniani 2, 35121 Padova, Italy; 2 Istituto Oncologico Veneto, U.O. Oncologia Medica 2, Via Gattamelata 64, 35128 Padova, Italy; 3 Istituto Europeo di Oncologia, Div. Chirurgia Toracica, Via Ripamonti 435, 20141 Milan, Italy; 4 Istituto Europeo di Oncologia, Div. Oncologia Medica, Via Ripamonti 435, 20141 Milan, Italy; 5 Istituto Nazionale Tumori “Regina Elena”, Div. Oncologia Medica, Via Chianesi 53, 00144 Rome, Italy; 6 Ospedale dell’Angelo, Div. Oncologia Medica, Via Paccagnella 11, 30174 Mestre, (VE), Italy; 7 Eli Lilly Italia, Via Gramsci 731/733, 50019 Sesto, Fiorentino, (FI), Italy; 8 Eli Lilly UK, Erl Wood Manor, Windhlesham, Surrey GU20 6PH, United Kingdom (former employee; 9 Istituto Nazionale Tumori “Regina Elena”, Div. Chirurgia Toracica, Via Chianesi 53, 00144 Rome, Italy

**Keywords:** Pemetrexed, Pleural mesothelioma, Chemotherapy, Surgery, Radiation

## Abstract

**Background:**

Malignant pleural mesothelioma is an aggressive tumor that has a poor prognosis and is resistant to unimodal approaches. Multimodal treatment has provided encouraging results.

**Methods:**

Phase II, open-label study of the combination of chemotherapy (pemetrexed 500 mg/m^2^+cisplatin 75 mg/m^2^ IV every 21 days × 3 cycles), followed by surgery (en-bloc extrapleural pneumonectomy, 3–8 weeks after chemotherapy) and hemithoracic radiation (total radiation beam 54 Gy, received 4–8 weeks post-surgery). The primary endpoint was event-free survival, defined as the time from enrollment to time of first observation of disease progression, death due to any cause, or early treatment discontinuation.

**Results:**

Fifty-four treatment-naïve patients with T1-3 N0-2 malignant pleural mesothelioma were enrolled, 52 (96.3%) completed chemotherapy, 45 (83.3%) underwent surgery, 22 (40.7%) completed the whole treatment including 90-day post-radiation follow-up. The median event-free survival was 6.9 months (95%CI: 5.0-10.5), median overall survival was 15.5 months (95%CI 11.0-NA) while median time-to-tumor response was 4.8 months (95%CI: 2.5-8.0). Eighteen (33.3%) and 13 (24.1%) patients were still event-free after 1 and 2 years, respectively. The most common treatment-emergent adverse events were nausea (63.0%), anemia (51.9%) and hypertension (42.6%).

Following two cardiopulmonary radiation-related deaths the protocol was amended (21 [38.9%] patients were already enrolled in the study): the total radiation beam was reduced from 54 Gy to 50.4 Gy and a more accurate selection of patients was recommended.

**Conclusions:**

The combination of pemetrexed plus cisplatin followed by surgery and hemithoracic radiation is feasible and has a manageable toxicity profile in carefully selected patients. It may be worthy of further investigation.

**Trial registration:**

Clinicaltrial.com registrationID #NCT00087698.

## Background

Malignant pleural mesothelioma (MPM) is an aggressive tumor originating from the superficial serosal cells of pleural cavities that has long been considered rare. Indeed, its incidence has considerably increased over the past two decades in industrialized countries on account of the widespread use of asbestos, the main carcinogen involved in its pathogenesis. The incidence of malignant mesothelioma is currently reported to range from 7 to 40 cases per million people, but it is believed that it will increase in the next 10 to 15 years [1].

MPM rapidly spreads to adjacent structures of the thoracic cavity. This explains why the majority of patients present with locally advanced or metastatic disease: 48% are in stage III and 40% are in stage IV [2,3].

The prognosis of MPM is poor. The median overall survival (OS) from diagnosis is 12 months, ranging from 8 months in stage IV patients to 40 months in stage I patients [4].

At present, an optimal therapy for MPM has not been established. In the recent Mesothelioma and Radical Surgery (MARS) study, in which patients with pathologically confirmed mesothelioma were randomized to extrapleural pneumonectomy (EPP) or no EPP within the context of trimodal therapy, the surgery option was reported to offer no benefit and possibly harms patients [5]. Previous studies showed that MPM is refractory to a series of single modality regimens based on chemotherapy, surgery, or radiotherapy [6]. An attempt to curative surgery, consisting of EPP, is feasible in no more than 5% of subjects [2]. Single-agent chemotherapy provides poor response rates (<20%) and no improvement in median survival [4]. The only advancement with a single modality is the identification of cisplatin as the best anticancer agent for MPM in a meta-analysis of studies published between 1965 and 2001. The combination of cisplatin and pemetrexed has shown to improve the objective response rate and to prolong median overall survival of patients with MPM versus cisplatin alone (41% vs 16.7%, 12.1 vs 9.3 months) within the context of a prospective, randomized, phase III clinical trial [7,8]. Radiotherapy with curative intent is usually not recommended, as target volumes exceed normal tissue tolerance limits [9]. Even though the modern Intensity Modulated Radiation Therapy (IMRT) technique seems to provide additional treatment options, it still requires further investigation [4,7].

In view of the resistance of MPM to single modality approaches, multimodal therapeutic strategies have been proposed. Studies assessing trimodal treatment (induction chemotherapy, followed by EPP and post-operative radiotherapy) have provided encouraging results [10,11].

The present trial, whose primary endpoint is event-free survival (EFS), was designed to test the feasibility, and assess efficacy and safety of the combined modality approach (induction chemotherapy followed by EPP and hemithoracic radiation) in patients with MPM with clinical stage I, II, or III (T1-3 N0-2).

## Methods

**Figure 1:**
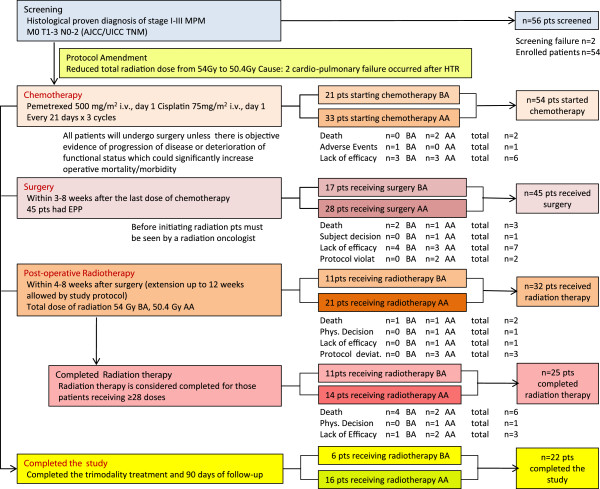
**Study design and patients disposition adopted in this study.** Study design highlights the trimodality therapy adopted: patients disposition is provided for patients enrolled before the protocol amendment (PA) as well as for those enrolled after PA and overall. Trimodality therapy was considered completed for those patients who received ≥28 doses of radiotherapy (RT) while the study was considered completed for those subjects completing RT and the 90-day post-radiation follow-up. pts = patients; MPM = malignant pleural mesothelioma; HTR = hemithoracic radiation; EPP = extrapleural pneumonectomy; BA = before amendment; AA = post amendment.

The present clinical trial (clinicaltrial.com ID #NCT00087698) aimed to assess the efficacy of the trimodality approach (chemotherapy, surgery and radiation) for the treatment of stage I-III MPM. A CONSORT (consolidated standard in reporting trials)-like diagram, including study design and patients disposition is presented in Figure 1. The study was approved by local ethics committees (Comitato Etico degli Istituti Fisioterapici Ospitalieri (IRCCS) di Roma, Rome, Italy; Comitato Etico Istituto Europeo di Oncologia, Milan, Italy; Comitato Etico Per La Sperimentazione Azienda Ospedaliera of Padua, Padua, Italy; Comitato Etico ULSS 12 Veneziana, Venice, Italy) and was conducted in accordance with the Declaration of Helsinki on ethical principles for medical research involving human subjects.

### Objectives of the study

The primary efficacy objective of this study was to assess the EFS, defined as the time from enrollment to the first observation of disease progression, death due to any cause, or early treatment discontinuation. EFS was censored at the date of the last follow-up visit for patients who did not discontinue early, who were still alive and had not progressed.

Secondary efficacy objectives were to assess: 1- and 2-year EFS rates; progression-free survival (PFS), defined as the time from study enrollment to the first date of disease progression or death as a result of any cause (PFS was censored at the date of the last follow-up visit for patients who were still alive and who have not progressed); OS, defined as the time from study enrollment to time of death from any cause (OS was censored at the date of last follow-up for patients who were still alive); and time to tumor response (TTR), defined as the time from study enrollment to the first observation of an objective tumor response.

### Eligibility criteria

Patients who were at least 18 years old, had signed informed consent and met all criteria listed in Table 1 were enrolled in the study.

**Table 1:** Eligibility criteria of this study


*Eligibility Criteria*	Clinical stage I, II or III (M0; N0-2; T1-3) pleural mesothelioma
	Performance status 0 to 1on the ECOG^a^ performance status schedule
	No previous surgical resection of mesothelioma
	No previous radiation therapy
	Estimated life expectancy of at least 12 weeks
	Adequate cardiac function
	Must be judged suitable to the therapy by medical oncologist and thoracic surgeon.
	Pulmonary function tests: FEV1^b^ >0.8, DLCO^c^ >35% of predicted postoperative FEV1 (ppoFEV1)
	ABG^d^ Predicted postoperative pCO2 <50.
	Adequate bone marrow reserve:
	· absolute neutrophil (segmented and bands) count (ANC) ≥1.5 × 10^9^/L,
	· Platelets ≥100 × 10^9^/L, and hemoglobin ≥9 g/dL.
	Hepatic: bilirubin ≤1.5 times the upper limit of normal (× ULN), alkaline phosphatase (AP), aspartate transaminase (AST) and alanine transaminase (ALT) ≤3.0 × ULN.
	Female patients of child-bearing potential must test negative for pregnancy at the time of enrollment based on a serum pregnancy test. Male and female patients must agree to use a reliable method of birth control during and for 3 months following the last dose of study drug.
	Geographical condition must not hamper the compliance with the study protocol and follow-up schedule.

^a^ Eastern Cooperative Oncology Group;^b^ Forced Expiratory Volume;^c^ Diffusing Capacity of the Lung for Carbon monoxide;^d^ Arterial Blood Gas.

### Treatment plan

#### Chemotherapy

Pemetrexed (500 mg/m^2^ intravenous [IV] infusion) followed by cisplatin (75 mg/m^2^ IV infusion) were administered to patients on Day 1 of a 21-day cycle for a total of three cycles. Supportive therapy of folic acid, vitamin B_12_ and dexamethasone (4 mg bid per os) were given.

#### Surgery

EPP was performed 3 to 8 weeks after the last dose of chemotherapy in eligible patients to achieve the complete resection of the gross residual tumor. EPP involved en-bloc resection of the pleura, lung, diaphragm, and ipsilateral half of the pericardium. Patients were eligible for surgery unless objective evidence of disease progression was present or deterioration of functional status occurred.

#### Radiation

Radiation was given to patients starting from 4 to 8 weeks after EPP (an extension to 12 weeks was possible as per study protocol). The clinical target volume (CTV) was contoured on each patient after a radiation oncologist and thoracic surgeon had discussed it. A total of 54 Gy radiation beam (30 × 1.8 Gy fractions) was administered to patients. Once patients received a total radiation of 40 Gy a non-enhanced CT scan was performed for safety evaluation. Due to two cardiopulmonary-related deaths that occurred earlier in the study, the study protocol was amended reducing the total radiation beam to 50.4 Gy (28 × 1.8 Gy fractions). Additional cardiopulmonary and respiratory function tests were also performed (Table 2).

**Table 2:** Summary of variation in selection criteria for cardiopulmonary and respiratory tests by protocol amendment

**Selection criteria**	**Before protocol amendment**	**After protocol amendment**
FEV 1	>2 L, or >35% of predicted postoperative (ppoFEV1); if < 2 L, the ppoFEV1 must be at least 35% based on the following formula using the quantitative V/Q scan: Predicted post-resection FEV1 = FEV1 x % perfusion to uninvolved lung from quantitative lung V/Q scan report.	> 0.8
DLCO	> 35% of ppoFEV1	
ABG	pCO_2_ < 50	predicted postoperative pCO2 < 50
Adequate cardiac function	The patient's cardiac function and presence or absence of coronary artery disease or valvular heart disease should be assessed by one of the following tests:	The patient's cardiac function and presence or absence of coronary artery disease or valvular heart disease should be assessed by the following tests:
	· Radionuclide stress test: preferred	· Echocardiogram (mandatory): Ventricular Ejection Fraction ^3^ 45%
	· Exercise stress test to maximal exercise level	· Electrocardiogram (mandatory)
	· Stress echocardiogram	· Radionuclide stress test (optional)
		· Exercise stress test to maximal exercise level (optional)

### Statistics

#### Determination of sample size

Based on the assumption of a 1-year EFS rate of about 30%, 53 patients were planned to be enrolled in the study to obtain a 95% confidence interval (CI) width of about ±12% around the 1-year EFS rate.

#### Efficacy analysis

Efficacy analyses were performed on the full analysis set (FAS), defined as all patients with histological or cytological diagnosis of MPM; no prior or concurrent systemic chemotherapy, immunotherapy, or biological therapy; presence of measurable or evaluable disease (for clinical response); and treated with at least one dose of pemetrexed/cisplatin. For all time-to-event variables, Kaplan-Meier curves were generated, quartiles and point probabilities (1- and 2-year time-to-event rates) were calculated and 95% CIs around the estimates were provided.

#### Safety analysis

Safety analyses were performed on the safety population, defined as all patients who received at least one dose of pemetrexed/cisplatin. Safety analyses included analyses of vital signs, laboratory and non-laboratory variables at each visit, and the changes from baseline. Shift tables showing laboratory test values classified as below normal, normal, and above normal, based on reference ranges, from baseline to each visit, were also produced.

## Results

The study was conducted at four centers in Italy from June 2005 to February 2010. A total of 56 patients were screened for the study and 54 were enrolled.

### Patient characteristics

The patients were mostly men (n=47, 87%) and had a median age of 63.0 years (range: 39–75 years) (Table 3).

**Table 3:** Characteristics of patients enrolled before and after the study protocol amendment

		**Before PA**^**a**^	**After PA**	**All**
*N Gender,* n (%)		21	33	54
	Male	17 (81.0)	30 (90.9)	47 (87.0)
	Female	4 (19.0)	3 (9.1)	7 (13.0)
*Age (yrs) Median (range)*		61.0 (39–71)	64.0 (44–75)	63.0 (39–75)
*Race,* n (%)				
	Caucasian	21 (100.0)	33 (100.0)	54 (100.0)
*Clinical Staging,* n (%)				
	I (T1,N0,M0)	1 (4.8)	11 (33.3)	12 (22.2)
	II (T2,N0,M0)	2 (9.5)	7 (21.2)	9 (16.7)
	III (T3,N1-2 M0)	17 (81.0)	16 (48.5)	33 (61.1)
*Histology,*n (%)				
	Epithelial	18 (85.7)	30 (90.9)	48 (88.9)
	Biphasic	2 (9.5)	1 (3.0)	3 (5.6)
	Desmoplastic	1 (4.8)	2 (6.1)	3 (5.6)

^a^ Protocol Amendment

Data are presented stratified by time of enrollment in this study with respect to the protocol amendment and are expressed as number of patients (percentage) except for age, expressed as mean (SD).

Twelve patients (22.2%) were in clinical stage I (T1, N0, M0), 9 (16.7%) were in clinical stage II (T2, N0, M0) and 33 (61.1%) were in clinical stage III (T1-3, N1-2, M0). Twenty-one patients (38.9%) were enrolled before the protocol amendment, implemented after two cardiopulmonary failure-related deaths, reducing the dose of radiotherapy from 54 Gy in 30 fractions to 50.4 Gy in 28 fractions.

### Treatment

#### Chemotherapy

Of the 54 patients enrolled, 52 (96.3%) patients completed pre-operative chemotherapy; one patient received only 2 cycles because of lack of efficacy and another received only one cycle because he experienced an acute myocardial infarction.

The median relative dose intensity of pemetrexed was 100% (range 33%-113%), that of cisplatin was 100.3% (range 33%-144%). Out of 159 cycles, 8 cycles of pemetrexed (5.0%) were delayed, one (0.6%) was reduced, and one (0.6%) was reduced and delayed, whereas 7 cycles of cisplatin (4.4%) were delayed, one (0.6%) was reduced, and two (1.3%) were delayed and reduced.

#### Surgery

Of the 54 patients enrolled, 9 patients dropped out of the study before surgery (6 due to lack of efficacy, 2 due to death and one patient was found to have invasion of the esophagus) while 45 (83.3%) underwent surgery. Of these, 9 patients were in clinical stage I, 8 were in clinical stage II and 34 were in clinical stage III. Among the patients who underwent surgery, 41 (91.1%) received EPP, while 4 (8.9%) did not. En- bloc EPP was performed in 36 patients (80.0%), non-“en- bloc” EPP in 5 patients (11.1%). Resection of the pericardium was performed in 38 patients (84.4%), resection of the diaphragm in 40 (88.9%) patients. A diaphragmatic prosthesis was inserted during 34 procedures (75.6%), a pericardial prosthesis in 33 patients (73.3%). A total of 2 patients (4.4%) died within 30 days of surgery.

#### Radiotherapy

Of the 45 patients who underwent surgery, 32 received radiotherapy (59.3% of total enrolled patients) while 13 (24.1%) did not (see Figure 1 for details). The median number of fractions received was 28 (range 12–30) and the median total dose received was 50.4 Gy (range: 21.6 to 54.0 Gy).

Twenty-two patients (40.7%) completed the trimodality treatment and the 90-day post radiation follow-up (Figure 1). Out of the remaining 32 patients, 16 were removed from the study earlier because of inefficacy (29.6%), mainly after chemotherapy or after surgery; 11 (20.4%) died, two (3.7%) violated the protocol, one experienced an unacceptable adverse event, one decided to withdraw, and one was removed from the study by the investigator. Out of the 11 deaths, five were considered to be unrelated to the study (diagnosis: bronchopneumonia, septic shock, suspected heart attack, underlying disease, suicide), two were due to study drug toxicity (cardiopulmonary failures), four were procedure related (1 empyema, 1 cardiac arrest, and 2 sepsis).

### Efficacy

**Figure 2:**
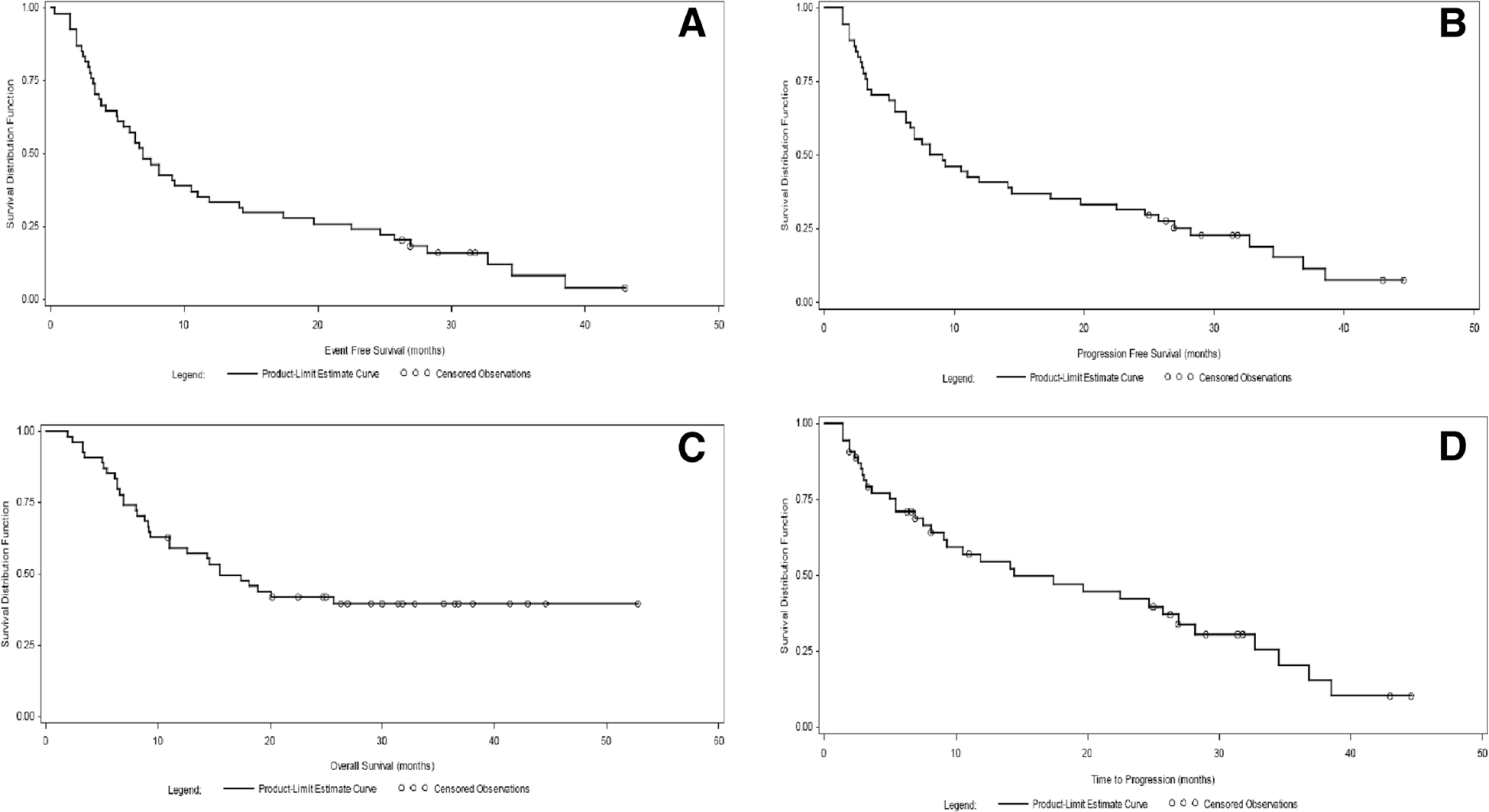
**Efficacy variables of this study.** Kaplan-Meier plots for efficacy variables analyzed for this study are presented: Median EFS (panel **A**), Median PFS (panel **B**), Median OS (panel **C**) and median TTR (panel **D**). Results are expressed in months. EFS= event-free survival; PFS= progression-free survival; OS= overall survival; TTR= time to tumor response.

The median EFS was 6.9 months (95%CI: 5.0- 10.5) (Figure 2A). A total of 18 (33.3%; 95%CI: 21.1- 47.5) and 13 (24.1%, 95%CI: 13.7- 36.0) patients were still event-free after 1 and 2 years, respectively. The median PFS was 8.6 months (95%CI: 6.3, 14.4 months) (Figure 2B); the one-year PFS rate was 40.7% (95%CI: 27.7-53.4); the 2-year PFS rate was 31.5% (95%CI: 19.7-43.9). The median OS was 15.5 months (95%CI: 11.0-NA) (Figure 2C). The 1-year OS rate was 59.2% (95%CI: 44.9- 70.9). Median TTR was 4.8 months (95%CI: 2.5-8.0) (Figure 2D). Overall, 31 patients had progression of disease. Of these, 10 patients had progression of disease during the study (site of progression: 6 local, 4 distant), while 21 had progression of disease during the follow-up period (site of progression: 5 local, 1 contralateral intrathoracic, 15 distant). Following two cardiopulmonary-related deaths, the study protocol was amended by reducing the total radiation dose administered to patients (see Methods section) and including additional cardiopulmonary and respiratory function tests (Table 2), which led to a more careful selection of patients being carried out.

### Safety

No important changes in vital signs, body weight, and Eastern Cooperative Oncology Group (ECOG) performance status were recorded.

Nearly all patients experienced at least one adverse event (AE) (n=53, 98.1%). The most common treatment-emergent AEs were nausea (63.0%), anemia (51.9%) and hypertension (42.6%). The total number of deaths recorded was 32 patients (59.3%). Of these, 11 patients died during the study (causes of death were: 1 broncopneumonia, 1 septic shock, 1 empyema, 1 suspect heart attack, 1 cardiac failure, 1 respiratory failure, 1 cardiac arrest, 2 sepsis, 1 suicide and 1 death for unknown causes) while 21 died during the follow-up period (causes of death were: 19 progression of disease [10 distant vs 9 local], 1 sepsis, 1 unknown).

Twenty-six patients (48.1%) required a blood transfusion. Thirty-six (66.7%) patients experienced at least one grade 3/4 toxicity and two patients died because of cardiopulmonary failure. Forty-two patients (77.8%) experienced at least one AE related to chemotherapy, which in 5 patients (9.3%) were serious. The corresponding rates for radiotherapy were 48.1% and 13%, respectively. The number of episodes of grade 3/4 toxicities related to treatment were: lymphopenia (n=3), neutropenia (n=3), dyspnea (n=3, all serious), anemia (n=2, 1 serious), heart failure (n=2, both serious), respiratory failure (n=2, both serious), acute cor pulmonale, myocardial infarction, pericardial effusion, emphysema, acute respiratory distress syndrome, pneumonia, bronchial fistula, subcutaneous emphysema (n=1 each, all serious), dysphagia (n=1), mucosal inflammation (n=1), hyponatremia (n=1), and scar pain (n=1). No important qualitative differences were recorded after the implementation of protocol amendment.

## Discussion

Malignant pleural mesothelioma is an aggressive tumor refractory to single regimens based on chemotherapy, surgery, or radiation [5]. Although IMRT technique seems to provide an additional treatment option (still requiring further investigations) clinical studies provided encouraging results on combined modality approach treatments [10,11]. The present study was designed to test the feasibility and assess the efficacy and safety of the combination of pemetrexed and cisplatin followed by EPP and hemithoracic radiation.

For the primary endpoint of EFS assessed for the study, the median EFS was 6.9 months (95% CI: 5.0 to 10.5), while the 1- and 2-year EFS rates were, respectively, 33.3% (95% CI: 21.1- 47.5) and 24.1% (95% CI: 13.7- 36.0). Considering that the sample size determination was based on the assumption that the expected 1-year EFS rate of about 30% (with a 95% CI of about ±12%), the 1-year EFS rate observed in the present study aligned with the initial assumption. Therefore, these results indicate that the adopted multimodality approach is feasible.

Although the 1-year EFS rate observed based on all the enrolled patients in the present study aligns with what is expected, it is possible that the subgroup of patients who completed all the three treatments have higher survival benefits. Indeed, a total of 22 (40.7%) of the 54 patients completed all the study treatments including the 90-day post-radiation follow-up. However, given the change in patients’ selection criteria following the protocol amendment (as described in the Methods section), it is important to consider mainly results based on all enrolled patients rather than the subgroup of patients in the present study.

Generally, results obtained for efficacy endpoints other than EFS, were lower than those reported by Van Schil et al. [12] in the EORTC study, a clinical trial with similar study design but different primary objective. The median PFS and 1-year PFS rate were 8.6 months (95% CI: 6.3- 14.4) and 40.7% (95% CI: 27.7-53.4), respectively, compared to 13.9 months (95% CI: 10.9-17.2) and 54.4% (95% CI: 40.7-66.2), respectively, obtained in the EORTC trial [12]. The median OS was 15.5 months (95% CI: 11.0-NA) compared to 18.4 months (95% CI: 15.6-32.9) in the EORTC [12]. The same outcome in the MARS trial was of 14.4 months (95%CI: 5.3-18.7) for patients randomized to EPP and of 19.5 months (95%CI: 13.4-not reached) for patients randomized to no EPP. [5]. Furthermore, the efficacy outcomes of the present study were less favorable as compared with those obtained in the SAKK and USA phase II trials [9,10].

However, we have to consider that following two cardio-pulmonary related deaths the study protocol was amended and the total radiation dose was reduced from 54 Gy to 50.4 Gy. It is noteworthy that the outcomes of efficacy endpoints, including the primary endpoint of EFS, did not worsen but instead seem to benefit from the changes introduced by the protocol amendment. Even though no claim of statistical significance can be made against the variation of efficacy variables when measured before or after the amendments, these findings seem to suggest that the reduced post-surgery radiation dose may lead to increased OS, EFS, PFS and TTR (Table 4) but this hypothesis needs to be confirmed in a further study. With regard to the safety profile, the adopted combination of pemetrexed plus cisplatin followed by EPP and hemithoracic radiation did not result in abnormal values either for cardiac or pulmonary function tests at baseline and after surgery. These results, taken together with no clinically relevant changes in vital signs, no substantial changes in the blood chemistry from baseline and generally acceptable decreases in certain hematologic values (hemoglobin, red blood cells [RBCs] count, white blood cells [WBCs] count, platelets and neutrophils), indicate a manageable toxicity profile following the reduced radiation dose.

**Table 4:** Summary results for efficacy variables

		**Before PA**^**a**^	**After PA**	**Overall**
*EFS*^b^				
	Median	6.3 (3.3-10.5)	8.1 (4.1 - 19.7)	6.9 (5.0-10.5)
	1-year Rate	23.8 (8.2-47.2)	39.4 (22.9-57.9)	33.3 (21.1-47.5)
	2-year Rate	14.3 (3.6-32.1)	30.3 (15.9-46.1)	24.1 (13.7-36.0)
*PFS*^c^				
	Median	6.6 (5.0-11.0)	11.9 (6.9-26.9)	8.6 (6.3-14.4)
	1-year Rate	28.6 (11.7-48.2)	48.5 (30.8-64.1)	40.7 (27.7-53.4)
	2-year Rate	19.1 (5.9-37.7)	39.4 (23.1-55.4)	31.5 (19.7-43.9)
*OS*^d^				
	Median	12.6 (6.9-21.1)	NA	15.5 (11.0-NA)
*TTR*^e^				
	Median	2.8 (1.4-5.1)	7.6 (4.1-8.9)	4.8 (2.5-8.0)

^a^ Protocol Amendment; ^b^ Event-free Survival; ^c^ Progression-free Survival; ^d^ Overall Survival; ^e^ Time to Tumor Response.

Overall results are presented as well as results stratified by time of enrollment in the study with respect to the protocol amendment. Results are expressed in months (95% CI) for median variables and as percentage (95% CI) for 1- and 2-year rates.

Unlike the MARS trial [5] that was designed to assess the clinical outcomes of patients who were randomly assigned to EPP or no EPP in the context of trimodal therapy, the present non-randomized study, that was disegned to to test the feasibility and assess the efficacy and safety of the combination of pemetrexed and cisplatin followed by EPP and hemithoracic radiation, recruited also patients of worst outcome (i.e. desmoplastic MPM histotype – see also Table 3). However, the low number of patients belonging to this subpopulation may not have had an impact on the final results. Moreover, the MARS trial allowed three different regimens of chemotherapy at physician discretion [5] while the present trial considered a pemetrexed-based regimen as the only chemotherapy option.

Based on median survival (14.4 months (95%CI: 5.3-18.7) for patients randomized to EPP and of 19.5 months (95%CI: 13.4-not reached) for patients randomized to no EPP) and mortality (18%) outcomes recorded in the MARS trial, Treasure et al. [5], concluded that EPP within trimodality therapy offers no benefit and possibly harms patients. As suggested by Weder et al. [13] this conclusion is not supported by data and possibly might move clinical research for mesothelioma in the wrong direction [13]. Provided that superiority of pleurectomy vs EPP has not been proved yet, we would like to strongly remark that the present study was not designed to compare different surgical strategies, therefore no conclusion nor speculation about pleurectomy vs EPP can be done. On the other hand, the low rate of completion (40.7%) recorded in this study, highlight the need for a careful patient selection and eventually, for a tailored surgical and medical approach.

The present study has limitations that were mainly introduced by the protocol amendment even though not predictable in advance. Because improvement of efficacy outcomes was not expected a priori, the sample size was not recalculated to allow comparison of data stratified by time of enrollment with respect to the protocol amendment. Thus, the analyses were limited to the overall efficacy outcomes. A possible additional bias may be that, after the protocol amendment, patients were not equally enrolled across all the participant study centers.

## Conclusions

This study provides further evidence that, under a careful patient selection, the combination of pemetrexed plus cisplatin followed by EPP and hemithoracic radiation is feasible, with a manageable toxicity profile, and may be further exploited.

## Abbreviations

AE: Adverse Event; CI: Confidence Interval; CONSORT: Consolidated Standard of Reporting Trials; CTV: Clinical Target Volume; ECOG: Eastern Cooperative Oncology Group; EFS: Event Free Survival; EORTC: European Organization for Research and Treatment of Cancer; EPP: Extrapleural Pneumonectomy; IMRT: Intensity Modulated Radiation Therapy; MARS: Mesothelioma and Radical Surgery; MPM: Malignant Pleural Mesothelioma; NA: Not Assessable; OS: Overall Survival; PFS: Progression Free Survival; SAKK: Swiss Group for Clinical Cancer Research; TTR: Time to Tumor Response.

## Competing interests

The authors declare the following conflict of interest: FR, AF, GM, LS, TMDP, AC, AP, GC and FF declare no conflict of interest. FR, MC and PM are full time employees of Eli Lilly Italy. GK was a contractor employee of Eli Lilly UK. This study was fully sponsored by Eli Lilly Italy, Sesto Fiorentino, Florence, Italy.

## Authors’ contributions

FF is the principal investigator, has supervised the study and has contributed to study design, interpretation of data and has substantially revised the manuscript. FR, GM, AF, LS, TMDP, AC, AP, GC and FR have contributed to acquisition of data, data interpretation and content review. MC has contributed to acquisition of data, data analysis, data interpretation and content review. GK has contributed to data analysis, data interpretation and content review. PM has contributed to data analysis, data interpretation, manuscript writing and content revision. All authors read and approved the final manuscript.

## Pre-publication history

The pre-publication history for this paper can be accessed here:

http://www.biomedcentral.com/1471-2407/13/22/prepub
